# The Use of Toll-Like Receptor Agonists in HIV-1 Cure Strategies

**DOI:** 10.3389/fimmu.2020.01112

**Published:** 2020-06-11

**Authors:** Janne Tegder Martinsen, Jesper Damsgaard Gunst, Jesper Falkesgaard Højen, Martin Tolstrup, Ole Schmeltz Søgaard

**Affiliations:** Department of Infectious Diseases, Aarhus University Hospital, Aarhus, Denmark

**Keywords:** HIV-1, TLR, innate immunity, HIV-1 cure, HIV-1 vaccine, latency reversing agents, immune modulation

## Abstract

Toll-like receptors (TLRs) are a family of pattern recognition receptors and part of the first line of defense against invading microbes. In humans, we know of 10 different TLRs, which are expressed to varying degrees in immune cell subsets. Engaging TLRs through their specific ligands leads to activation of the innate immune system and secondarily priming of the adaptive immune system. Because of these unique properties, TLR agonists have been investigated as immunotherapy in cancer treatment for many years, but in recent years there has also been growing interest in the use of TLR agonists in the context of human immunodeficiency virus type 1 (HIV-1) cure research. The primary obstacle to curing HIV-1 is the presence of a latent viral reservoir in transcriptionally silent immune cells. Due to the very limited transcription of the integrated HIV-1 proviruses, latently infected cells cannot be targeted and cleared by immune effector mechanisms. TLR agonists are very interesting in this context because of their potential dual effects as latency reverting agents (LRAs) and immune modulatory compounds. Here, we review preclinical and clinical data on the impact of TLR stimulation on HIV-1 latency as well as antiviral and HIV-1-specific immunity. We also focus on the promising role of TLR agonists in combination strategies in HIV-1 cure research. Different combinations of TLR agonists and broadly neutralizing antibodies or TLRs agonists as adjuvants in HIV-1 vaccines have shown very encouraging results in non-human primate experiments and these concepts are now moving into clinical testing.

## Introduction

Human immunodeficiency virus type 1 (HIV-1) infection can today be completely suppressed by antiretroviral therapy (ART). However, during the early phase of primary HIV-1 infection, the virus establishes a reservoir in infected long-lived immune cells, which necessitates life-long ART in order to prevent disease progression (1, 2). This latent HIV-1 reservoir is predominantly found in long-lived memory CD4+ T cells in which the provirus is transcriptionally silent and thus go undetected by the host immune system (3, 4). These proviruses can be (re)activated and go on to transcribe and form infectious particles, leading to re-emergence of active infection if ART is stopped. The HIV-1 reservoir is believed to be maintained through the long-lived nature and homeostatic proliferative capabilities of the latently infected memory T cells (5, 6).

The innate immune system constitutes a vital part of the early defense against infections and in controlling established HIV-1 infection. Therefore, strategies aimed at boosting innate immunity have gained great interest in HIV-1 cure research. Endogenous interferon production induced by activation of Toll-like receptors (TLRs) is one of the innate immune'system's first antiviral responses upon infection. TLRs are in the family of pattern recognition receptors (PRRs) which detect pathogen-associated molecular patterns (PAMPs) (7, 8). TLRs also respond to signs of host cell damage through ligands called damage-associated molecular patterns (DAMPs) (9).

TLRs are expressed on many different immune cells including natural killer (NK) cells, macrophages, B cells, and to a high degree on dendritic cells (DCs) (10) (Table 1). TLR1, TLR2, TLR4, TLR5, TLR6, and TLR10 are expressed on the cell surface, whereas TLR3, TLR7, TLR8, and TLR9 are located in the membrane of the intracellular endosomes (8, 14, 20). This partition in localization reflects the disparate pathogen-sensing function of these two groups. The cell surface-associated TLRs are mainly responsible for detecting components from extracellular microbes such as bacteria and fungi, whereas the TLRs in the endosomal compartment mainly detect virus and intracellular bacteria (18, 22, 23) (Table 1). TLRs are transmembrane proteins consisting of three different domains: an ectodomain consisting of leucine-rich-repeats (LRR) mediating ligand recognition, a transmembrane domain, and the cytosolic domain Toll/IL-1R (TIR), which mediates downstream signaling (18). The cytosolic domain can, upon activation of the TLR, recruit different domain-containing adaptor molecules such as myeloid differentiation primary-response protein 88 (MyD88), TIR-domain containing adaptor protein (TIRAP)] also called MyD88 adaptor-like (MAL), TIR-domain containing adaptor protein inducing IFN-β (TRIF), and TRIF-related adaptor molecule (TRAM) (23–25). The MyD88 signaling pathway used by all of the TLRs, except for TLR3, activates nuclear factor-κB (NF-κB) and mitogen-activated protein kinases (MAPKs) leading to the production of pro-inflammatory cytokines. The TRIF–dependent pathway used by TLR3 and TLR4 leads to induction of type 1 interferons (IFNs) in addition to pro-inflammatory cytokines (18, 20, 23). The cytokine induction initiated by TLR activation not only triggers an innate immune response, but also takes part in initiating and shaping the adaptive immune response (22, 26).

**Table 1:** A schematic outline of TLR localization, expression on immune cells, receptor complex formations, ligands, recruited TIR domain-containing adaptor molecules, and cytokine outcomes.

**TLR type**	**Cell type**	**Receptor-complex**	**Ligand**	**TIR-adaptor**	**Outcome**
**(A) TLRs on cell surface**					
TLR1	Monocytes DCs T cells	1–2	- Lipopeptides from bacteria and mycobacteria	MyD88	Pro-inflammatory cytokines
TLR2	Monocytes Macrophages DCs	2–1 2–2 2–6 2–10	- Components from the cell wall of gram-positive bacteria - Glycoprotein from virus - Zymosan from fungi	MyD88	Pro-inflammatory cytokines
TLR4	Monocytes DCs	4-MD2	- Lipopolysaccharides from gram-negative bacteria - Envelope components of respiratory syncytial virus	MyD88 TRIF TRAM TIRAP/MAL	Pro-inflammatory cytokines IFNs
TLR5	Monocytes T cells	5–5	- Flagellin from flagellated bacteria	MyD88	Pro-inflammatory cytokines
TLR6	Monocytes Macrophages B cells	6–2	- Lipopeptides from Mycoplasma	MyD88	Pro-inflammatory cytokines
TLR10	B-cells	10–2	- Ligands from Listeria - Ligands from Influenza A		
**(B) TLRs in endosomal compartments**					
TLR3	DCs	3-3	- Viral double-stranded RNA - Self-RNA from damaged cells	TRIF	Pro-inflammatory cytokines IFNs
TLR7	pDCs B cells	7-7	- Viral single-stranded RNA	MyD88	Pro-inflammatory cytokines IFNs^*^
TLR8	Monocytes DCs	8-8	- Viral single-stranded RNA - Bacterial RNA	MyD88	Pro-inflammatory cytokines
TLR9	pDCs B cells	9-9	- CpG containing DNA from bacteria and virus - Self-DNA from damaged cells - Hemozoin from Plasmodium Falciparum	MyD88	Pro-inflammatory cytokines IFNs^*^

* *IFNs through MyD88*.

*The table is a summary of TLR properties described previously (11–20). It should be noted that some immune cells subsets and ligands have been left out for the sake of overview and relevance to the subjects at hand. TLR expression on epithelial cells have been left out of Table 1 as well, but have been reviewed elsewhere (21)*.

The present review focuses on the emerging potential of synthetic TLR agonist treatment in HIV-1 cure research both alone and in combination with other interventions.

## TLR Agonists as Latency Reversing Agents (LRA)

The latent HIV-1 reservoir constitutes the main barrier to a cure for HIV-1 infection (27, 28). One of the proposed strategies toward overcoming this obstacle is the “shock and kill” approach (28, 29). The hypothesis behind this strategy is that LRA administration will (re)activate HIV-1 transcription in latently infected cells, leading to expression of viral antigens on their surface. This will in turn expose infected cells to immune-mediated killing and/or apoptosis due to viral-cytopathic effects while concurrent ART prevents released virions from infecting other immune cells (30).

A widely investigated group of LRAs is the histone deacetylase inhibitors (HDACi) including romidepsin, vorinostat, panobinostat, and chidamide (31–33). Inhibition of the histone deacetylase enzymes leads to a more accessible chromatin structure, thus enabling transcription of the latent proviral HIV-1 DNA (34, 35). While HDACi are now being investigated in combination with HIV-1 vaccines (36), broadly neutralizing antibodies (bNAbs) (www.clinicaltrials.gov: NCT03041012), and other LRAs, none of these HDACi alone have so far been capable of inducing a substantial reduction of the HIV-1 reservoir in clinical trials (37).

Protein kinase C (PKC) agonists such as bryostatin and prostratin (38) have shown significant latency reversal activity *ex vivo* but may be too toxic to be dosed at active concentrations in humans (39). However, the latency reverting effects of a natural plant extract containing ingenols, yet another group of PKC agonist, is currently under being tested in a clinical trial in HIV-1 infected individuals (NCT02531295). Disulfiram, a drug used for alcohol cessation, has also been tested in clinical trials as a potential LRA (40). Disulfiram induced increased levels of cell-associated unspliced HIV-1 RNA (usRNA) in study participants of three different dosing groups, but it did not lead to significant changes in either total HIV-1 DNA or plasma levels of HIV-1 RNA. The latency reversing properties of other compounds such as cytokines and other epigenetic modifiers have similarly been investigated (41, 42). However, the discovery of a single therapeutic capable of inducing significant HIV-1 reservoir alterations is still to be made.

The first TLR agonist to draw attention to the potential utilization of TLR agonists as LRAs was that of the antisense oligodeoxynucleotide (ODN) phosphorothioate Gene-Expression Modulator 91 (GEM91). GEM91 was initially shown to inhibit HIV-1 replication *ex vivo* in human peripheral blood mononuclear cells (PBMCs) from HIV-1 infected donors (43). Unexpectedly, a subsequent GEM91 dose-escalation study showed increased viremia following administration in HIV-1 infected individuals contradictory of the *ex vivo* findings (44). It was later discovered that this potential induction of viremia was due to a CpG motif in GEM91 leading to TLR9 stimulation. Thus, it was proposed that the increased viremia was caused by innate immune activation and concomitant HIV-1 (re)activation (45–47).

Several TLR agonists have since been investigated as LRAs because of their ability to induce immune activation, and in doing so, causing (re)activation of silent HIV-1 in latently infected cells and boosting the antiviral immune response. These mechanisms are elaborated in the section “Immunomodulatory properties of TLR agonists.”

### *Ex vivo* Experiments

Utilizing the optimal cell model for assessment of latency reversal is of great importance and should consider the type of LRA investigated. The majority of *in vitro/ex vivo* LRA experiments focuses on latently infected T cell lines or primary T cells. These cell models work well when investigating LRAs such as HDACi which (re)activate HIV-1 transcription by a direct impact on the target cell. Yet, most TLRs are not expressed at physiological levels on CD4+ T cells, which is why this lymphocyte subset is often unresponsive to direct TLR stimulation (e.g., by TLR7 or TLR9 agonists) (Table 1). Instead, these TLR agonists induce HIV-1 transcription indirectly through activation of innate immune cells such as DCs (Table 2) (18, 22, 23, 54).

**Table 2:** Schematic overview of *ex vivo* experiments investigating the utility of TLR agonists as LRAs included in the manuscript.

**References**	**TLR agonist**	**Cell type**	**Study design**	**Endpoint**	**Results**
Novis et al. (48)	1) 1/2, 2/6, 3, 4, 5, 7, 7/8, 9	1) T_CM_ cell model	1) *In vitro* stimulation	1) Intracellular p24 Gag protein expression	1) Only the TLR1/2 agonist induced significant increase (but the T_CM_ cell model does not express most TLRs)
	2) 1/2	2) T_CM_ from aviremic HIV-1 infected donors	2) *Ex vivo* stimulation	2) Intracellular level of usRNA	2) Significant increase in 2 of 7 donor samples (5 of 7 for panobinostat)
Kaur et al. (49)	1) 1/2, 3, 4, 5, 7, 8	1) PBMCs from aviremic HIV-1 infected donors	1) *Ex vivo* stimulation	1) Supernatant HIV-1 RNA level	1) All TLR agonists induced significant increase
	2) 1/2, 3, 4, 5, 7, 8	2) CD4+ T cells from aviremic HIV-1 infected donors	2) *Ex vivo* stimulation	2) Supernatant HIV-1 RNA level	2) Only TLR ½ agonist Pam3CSK4 induced significant increase
Thibailt et al. (50)	1) 5	1) Resting T_CM_ from healthy donor PBMCs, infected with luciferease-encoding pseudotyped HIV-1	1) *Ex vivo* stimulation	1) HIV-1 long terminal repeat-driven luciferase activity	1) Increase
	2) 5	2) resting CD4+ T cells from aviremic HIV-1 infected donors	2) *Ex vivo* stimulation	2) Intracellular p24 Gag protein level	2) No increase
Tsai et al. (51)	1) 7	1) PBMCs from aviremic HIV-1 infected donors	1) *Ex vivo* stimulation	1) Supernatant mean HIV-1 RNA levels	1) Increase
	2) 7	2) PBMCs from aviremic HIV-1 infected donors, treated with antibodies against IFNAR on T cells	2) *Ex vivo* stimulation	2) Supernatant mean HIV-1 RNA levels	2) No increase
	3) 7	3) CD4+ T cells from aviremic HIV-1 infected donors	3) *Ex vivo* stimulation	3) Supernatant mean HIV-1 RNA levels	3) No increase
Bam et al. (52)	1) 7	1) PBMCs from healthy donors	1) Pre-stimulating with GS-9620 for 2 days prior to infection with a luciferase reporter virus containing HIV-1	1) HIV-1 replication	1) Inhibition
	2) 7	2) CD4+ T cells from healthy donors	2) Pre-stimulating with GS-9620 for 2 days prior to infection with a luciferase reporter virus containing HIV-1	2) HIV-1 replication	2) No inhibition
Offersen et al. (53)	1) 9	1) PBMCs from aviremic HIV-1 infected donors	1) *Ex vivo* stimulation	1a) Level of usRNA in CD4+ T-cells extracted from PBMCs post stimulation	1a) Increase
				1b) Level of IFN-α in cell supernatant	1b) Increase

*The studies all have several endpoints in their study setup. The most relevant endpoints in the setting of this review have been outlined as (1), (2), and (3) for overview*.

This issue was highlighted in a study by Novis et al. who tested a broad panel of TLR agonists as LRAs (48). In a central memory CD4+ T cell (T_CM_) model, they found that only the TLR1/2 agonist Pam3CSK4 was able to significantly increase HIV-1 transcription, measured by intracellular p24 Gag protein expression, after stimulating the cells for 3 days (48). Subsequently, the latency reversing properties of Pam3CSK4 were tested *ex vivo* utilizing T_CM_ isolated from aviremic HIV-1 infected donors. They found that HIV-1 transcription increased in two out of seven donors after Pam3CSK4 stimulation compared to five out of seven after panobinostat stimulation, measured by the level of usRNA. Pam3CSK4 was demonstrated to (re)activate HIV-1 transcription via an NF-κB and NFAT-dependent pathway, but without induction of IFNs, thereby avoiding T cell activation (CD69+ and CD25+) and proliferation (cell proliferation dye assessment). However, the degree of HIV-1 (re)activation induced by Pam3CSK4 was lower than that of panobinostat.

In 2018, Kaur et al. conducted a comprehensive investigation of the LRA properties of agonists of 6 different TLRs(1/2, 3, 4, 5, 7, and 8) on both PBMCs and isolated CD4+ T cells from HIV-1 infected donors on long term ART (49). All TLR agonists were able to induce a modest but statistically significant fold-increase of supernatant HIV-1 RNA from PBMCs compared to vehicle controls. In isolated CD4+ T cells however, only the TLR1/2 agonist Pam3CSK4 was able to significantly increase the supernatant HIV-1 RNA level. These findings are in line with the findings of Novis et al. and indicate TLR1/2 expression on CD4+ T cells.

Thibault et al. tested the direct HIV-1 latency reversing properties of the TLR5 agonist flagellin on T_CM_ (50), which in a previous study showed indications of TLR5 expression (55) (Table 1). Resting T_CM_ isolated from healthy donor PBMCs were infected with luciferease-encoding pseudotyped HIV-1 particles. When treated with flagellin, the isolated T_CM_ showed enhanced HIV-1 gene expression compared to mock-treated controls, assessed by fold increase of HIV-1 long terminal repeat-driven luciferase activity. However, when flagellin stimulation was tested on resting CD4+ T-cells from virally suppressed HIV-1 infected donors, no induction of intracellular p24 Gag protein was detectable. The results of Thibault et al. are in accordance with those of Novis et al. and Kaur et al., but raise uncertainty regarding the physiological effects of the previously indicated TLR5 expression on T cells.

Tsai et al. demonstrated that GS-9620, a TLR7 agonist, was capable of (re)activating latent HIV-1 in PBMCs from HIV-1 infected donors after 4 days of stimulation, measured as a 1.6-fold increase in mean HIV-1 RNA levels in the cell supernatant compared to vehicle-treated controls (51). This effect was mediated by activation of plasmacytoid dendritic cells (pDCs) leading to an IFN-driven CD4+ T cell activation (CD69+), which was demonstrated by the lack of HIV-1 RNA production when PBMCs were treated with antibodies against interferon-α receptors (IFNAR) on T cells. No changes in supernatant HIV-1 RNA was found when performing direct stimulating of pure CD4+ T-cells, which is consistent with the above-mentioned inconsiderable TLR7 expression on human T-cells. In corroboration with these findings, Bam et al. demonstrated inhibition of HIV-1 replication in PMBCs pre-stimulated with GS-9620 for 2 days prior to infection with a luciferase reporter virus containing HIV-1 (52). This antiviral effect was not observed when isolated CD4+ T cells or macrophages were equivalently pre-stimulated.

As briefly described above, synthetic ODNs containing CpG motifs were the first version of the TLR9 agonists and were mainly investigated in the context of cancer treatment (56). The structurally different TLR9 agonists such as MGN1703 were later developed to improve the tolerability of the drug class (57). Our group investigated the “shock and kill” properties of the TLR9 agonist MGN1703 on PBMCs from HIV-1 infected individuals on ART (53). MGN1703 stimulation led to increased levels of usRNA in CD4+ T-cells after 16 h. This effect was mediated indirectly by activated pDCs secreting IFNs, inducing T-cell activation (CD69+). MGN1703 did however not increase the usRNA levels to the same extent as panobinostat.

Lastly, other studies have also suggested that agonists of TLR2/7 and TLR8 possess latency reversing properties *ex vivo* and *in vivo* (58, 59). Extensional investigation of latency reversing properties of TLR agonists utilizing cell lines and mouse models have been reviewed elsewhere (25, 60, 61).

The findings of the preclinical studies presented here suggest that while some TLRs are present on T cells, the physiological effect of TLR-mediated (re)activation of latent HIV-1 in transcriptionally quiescent memory T cells occur in large by means of DC activation and subsequent IFN-α stimulation of T cells. Therefore, the full immune stimulating or latency reversing effect of synthetic TLR agonists can often not be evaluated by utilizing single immune cell models (Table 2).

### Non-Human Primate (NHP) Studies

Evaluating pre-clinical *in vivo* studies investigating the therapeutic potential of TLR agonists in HIV-1 cure research, the present review will be focusing on NHP studies, as the TLR expression on immune cells of NHPs bare the closest resemblance to that of human immune cells, compared to smaller animal models (62, 63).

Of the TLR agonists investigated *ex vivo* as described above, only agonists of TLR3, TLR7, and TLR9 have progressed into clinical testing as potential LRAs in the field of HIV-1 cure research. However, TLR3 agonist testing quickly progressed from small animal models into clinical trials in advanced cancer disease and its LRA potential has not been assessed in NHPs (64–67). The majority of the preclinical work concerning TLR9 agonists have similarly been conducted in the field of cancer treatment and is thus beyond the scope of this review but has been described elsewhere (68).

Two different groups have investigated the potential of TLR7 agonists as LRAs *in vivo* in NHP. Lim et al. showed that the TLR7 agonist GS-986, was capable of inducing transient simian immunodeficiency virus (SIV) plasma RNA blips of up to 1,000 copies/mL, after 24 to 48 h (69). GS-986 was administered in escalating doses (0.1–0.3 mg/kg) by oral gavage and led to SIV RNA blips in 4 out of 4 virally suppressed rhesus macaques on ART, compared to 0 of 6 vehicle-treated controls. The blips were seen after dose 4, 5, 6, and 7, but not after the first 3 doses, which could indicate a priming effect of the TLR agonist. T, NK, and B cells were transiently activated within 24 to 48 h following stimulation and then returned to baseline. In the TLR7 agonist treated NHPs, the viral reservoir was reduced by an average of 75% as measured by total SIV DNA in memory CD4+ T cells [isolated from PBMCs, mononuclear cells from lymph node (LNMCs) and gastrointestinal mucosa (GMMCs)] compared to no change in total SIV DNA among control animals. However, there was no difference in time to viral rebound between the GS-986-treated group and the control group following ART cessation.

In a subsequent experiment, 9 NHPs, received 10 administrations of either GS-986 (0.1 mg/kg) or GS-9620 (0.05 or 0.15 mg/kg) followed by a 3-months resting period and then another 9 doses. 2 NHPs constituted a vehicle-control group. All TLR7 agonist-treated NHPs experienced blips in plasma SIV RNA levels during the first interventional period, but during the second interventional period, only 1 viral blip was measured, even though the activation level of T, NK and B cells was comparable between the two dosing periods. The reason for this variation is not clear but it could indicate increasing immune tolerance to TLR7 activation following repeated stimulation. Total SIV DNA was however again significantly reduced in both PBMCs and GMMCs in a combined analysis of all TLR7-agonists-treated groups compared to controls. In a modified viral outgrowth assay, the majority of TLR7-treated NHPs displayed a reduction in the inducible SIV reservoir. Two of the NHPs had no inducible SIV reservoir and showed durable control for more than 700 days after interruption of ART. Subsequent CD8+ T-cell depletion did not lead to emergence of virus, and neither did adoptive transfer of PBMCs and LMMCs to naïve macaques, suggesting a complete eradication of the viral reservoir.

Surprisingly, Del Prete et al. were unable to reproduce the findings of Lim et al. (70). Their study also included SIV-infected rhesus macaques that received 12 doses (0.15 or 0.5 mg/kg) of GS-9620, administered by oral gavage. No spikes in plasma SIV RNA levels were observed and no significant changes in transcriptional RNA/DNA ratios in PBMCs, LNMCs, or GMMCs were detected at 24 and 48 h post-dosing. All NHPs rebounded within 4 weeks upon ART cessation. CD4+ T cells showed no increased activation but at 24 h post-dosing CD8+ T cells had increased co-expression of CD38 and HLA-DR.

The cause of the difference in LRA effect and outcome in the two comparable NHP studies is unclear. In the study by Lim et al., ART was initiated on day 65 of infection and continued for around 437 days before the intervention, whereas Del Prete et al. initiated ART on day 13 and waited 525 days before intervening. The timing of ART initiation and/or of intervention might affect the viral reservoirs differently which could contribute to the discrepant findings. NHPs were infected intrarectally with SIVmac251 in the study of Lim et al., and intravenously with SIVmac239X in the study of Del Prete et al., which could also lead to varying reservoir properties.

Collectively, these findings indicate that the latency reversing effect of TLR7 agonists in NHPs might be very sensitive to alterations in model conditions such as timing of treatment initiation in relation to the course of infection, immune status, and SIV/SHIV challenge strain. The measured effects of the treatment may also depend greatly on the administered dose, dosing intervals, and timing of sampling.

### Clinical Trials

The first clinical trial investigating the effects of a TLR3 agonist in HIV-1 infected individuals on long-term ART was recently published by Saxena et al. (71). The synthetic double-stranded RNA polyinosinic:polycytidylic acid (poly-I:C) and its more stabilized form containing poly-L-lysine (poly-ICLC) have previously been tested in human cancer studies (67, 72). Saxena et al. tested poly-ICLC on 12 HIV-1 infected donors in doses of 1.4 mg administered subcutaneously once daily on two consecutive days. Follow-up measurements were obtained on day 4 and 8 and at week 4, 16, and 48. During the study period, poly-ICLC treatment did not affect the level of usRNA or total HIV-1 DNA in CD4+ T-cells. In addition, no significant changes in activation of DCs (CD40+CD83+CD86^+^) or T cells (HLA-DR+CD38+) were observed, except for a significant upregulation of CD38 expression on CD8+ T-cells at day 4, which normalized at day 8. The authors speculated that higher poly-ICLC doses, more frequent administration, or combinations with other therapeutics might be needed to achieve a robust immunological impact.

Assessing the safety of the TLR7 agonist GS-9620, Riddler et al. recently reported findings from a phase I dose escalation placebo-controlled study including 48 HIV-1 infected individuals on ART (NCT02858401) (73). In 6 treatment groups, GS-9620 doses ranging from 1 to 12 mg GS-9620 were administered every other week for up to 19 weeks. GS-9620 was generally well-tolerated, even in the 12 mg group, and there were no discontinuations due to adverse events. There was however no evidence of GS-9620 effectively impacting the latent HIV-1 reservoir as no consistent changes were observed in levels of plasma HIV-1 RNA, usRNA or total HIV-1 DNA in CD4+ T cells between the different groups. Another ongoing study with this compound is assessing the safety and efficacy of GS-9620 in ART-treated HIV-1 infected controllers, defined as individuals having a pre-ART viral load between 50 and 5,000 copies/mL (NCT03060447).

Since 2007, our group has been working with TLR9 agonists both as vaccine adjuvant and immune stimulator in HIV-1 infection. We conducted a pilot study in HIV-1 infected individuals whom received 60 mg of TLR9 agonist MGN1703, administered subcutaneously twice weekly over 4 weeks (74). During the 4-week intervention period, 6 of the 15 participants had detectable plasma HIV-1 RNA levels in the range of 21–1,571 copies/mL. This suggested a moderate latency reversing effect of MGN1703, but no reduction was observed in the proviral reservoir size assessed by total and integrated HIV-1 DNA in CD4+ T cells. No changes in levels of replication-competent proviruses were detected either. Both pDCs and T cells were activated as demonstrated by increased expression of the co-stimulatory markers CD40 and CD86 on pDCs and CD38 and HLA-DR on T cells. Surprisingly, the level of transcriptionally active CD4+ T cells, measured as cell-associated usRNA levels, decreased significantly during the 80-days follow-up period.

Based on these favorable immunological findings, an extension of the this study was conducted during which MGN1703 was administered twice weekly for 24 weeks to 12 HIV-1 infected participants (75). The prolonged intervention did, however, not reduce the size of the viral reservoir, assessed by measurements of total HIV-1 DNA or replication competent virus in a viral outgrowth assay. One participant (ID113) was able to control viral replication to undetectable levels for 150 days upon ART cessation. Immunological control in this individual was shown to be mediated in part by a superior level of HIV-1-specific CD8+ effector memory T cells, compared to the other study individuals. Of note, the IgG neutralization capacity of ID113 had increased during the MGN1703 treatment, supporting beneficial effect of the TLR agonist on adaptive immunity and in particular on B cell maturation (76).

Although several *ex vivo* and some *in vivo* studies have demonstrated encouraging results regarding the latency reversing effect of TLR agonists, there are no reproduceable data showing significant impact on the HIV-1 reservoir in clinical trials following TLR agonist treatment. A potential issue of LRAs as part of a HIV-1 cure strategy is that the focus is on latently infected CD4+ T cells as the main target of the latency reversal. While infected memory CD4+ T cells constitutes a long-lived HIV-1 reservoir, other cell types have been shown to harbor replication competent virus such as monocytes, macrophages, and dendritic cells (77, 78). Additionally, infected cells reside in other compartments than the blood including the lymph nodes and gut associated lymphoid tissue, and potentially also reside in the brain, the genital tract, and the lungs (79–84). However, the latter compartments are notoriously difficult to sample in clinical trials.

Collectively, based on the current knowledge we believe there is evidence for latency reversal following TLR (particularly TLR9) agonist treatment in HIV-1-infected individuals. However, TLR agonists' potency as LRAs appear to be relatively modest but as we outline below, certain TLR agonists are potent immune stimulators and it is in this capacity that they may have the biggest role to play in HIV-1 cure strategies.

## Immunomodulatory Properties of TLR Agonists

Upon TLR activation of DCs, proinflammatory cytokines such as IL-12 and IFNs are secreted, leading to auto- and/or paracrine activation of immune cells including other DCs, macrophages, NK cells, and T cells (85, 86). Activation of the DCs additionally leads to a downregulation of the inflammatory chemokine receptor CCR6 on the DC surface and upregulated expression of the lymphoid-homing receptor CCR7. This triggers DC migration from tissues to lymph nodes where they can present antigen to T and B cells and thus mediate an adaptive immune response (22, 87, 88). IFNα-α, produced mainly by pDCs activates both CD4+ and CD8+ T cells via their interferon alpha receptor (IFNAR) (51, 89, 90). Krieg et al. demonstrated a significantly increased frequency of antigen specific CD8+ T cells (0.07–3.00%) in 8 of 8 melanoma patients receiving a melanoma antigen vaccine adjuvanted with CpG ODNs compared to eight control patients receiving the vaccine alone (91). Accordingly, several clinical studies from our group have demonstrated HIV-1 (re)activation through an IFNα-α induced CD4+ T-cell activation and increased HIV-1-specific polyfunctionality of CD8+ T cells following TLR9 stimulation (74, 75, 92). Similar effects have been demonstrated *ex vivo* and *in vivo* in TL7TLR7 agonist studies (51, 93, 94).

**Figure 1:**
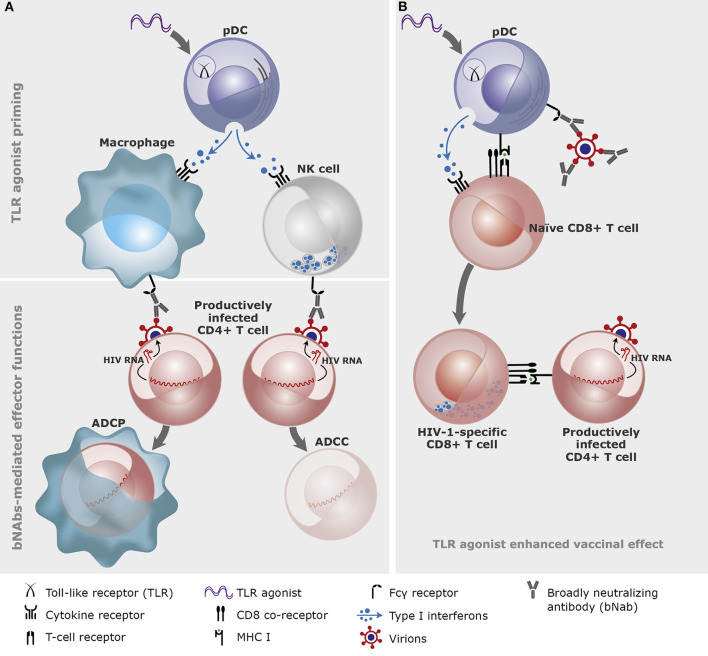
A conceptual illustration of the effects of Toll-like receptor (TLR) agonists and broadly neutralizing antibodies (bNAbs) in combination. **(A)** TLR agonist priming of innate immune cells through plasmacytoid dendritic cells (pDC). The primed innate immune cells (here depicted macrophage and natural killer (NK) cell) bind the broadly neutralizing antibodies via the Fcγ receptors and mediate antibody-dependent cellular phagocytosis (ADCP) or cytotoxicity (ADCC) of the productively infected CD4+ T cells. **(B)** TLR agonists and bNAbs-antigen complexes cross-prime CD8+ T cells. TLR agonists and bNAbs-antigen complexes bind to pDCs which cross-presents viral antigens on the MHC class I molecule to the naïve CD8+ T cells leading to development of HIV-1-specific CD8+ T cells (graphics: Gitte Skovgaard Jensen, AUH).

NK cells can mediate direct killing upon interaction with foreign pathogens or cells exhibiting signs of stress, but they are also the primary effector cells in mediating antibody-dependent cellular cytotoxicity (ADCC) via their Fcγ receptor (CD16) (26, 95). The cytotoxic effect is executed through the release of perforins which allows secreted cytotoxic granzymes to enter the targeted cell (96). The analogous anti-viral effector function of activated macrophages is that of phagocytosis, where the macrophage will devour entire infected cells (96). This effect can, like ADCC, be enhanced by antibodies and is thus called antibody-dependent cellular phagocytosis (ADCP). Both macrophages and NK cells are important in clearing HIV-1 infected cells (97, 98), and TLR agonists can thus work to increase their effector functions both directly through TLRs expressed on macrophages and NK-cells (Table 1) and indirectly via activation of B-cells and DCs (Figure 1).

Our group demonstrated enhanced NK cell function following *ex vivo* MGN1703-stimulation of PBMCs from HIV-1-infected individuals on long-term ART (53). The cytotoxic (CD56^dim^ CD16+) and cytokine-producing (CD56^bright^ CD16±) NK cells showed a significant 7.5- and 2.2-fold increase in CD69-expression, respectively, after 48 h of stimulation. Compared to unstimulated NK cells, MGN1703-stimulated NK cells additionally showed enhanced inhibition of HIV-1 production from autologous infected CD4+ T cells. Tsai et al. similarly demonstrated increased levels of activated NK cells (both CD56^dim^ CD16+ and CD56+ CD16-) upon GS-9620 stimulation of PBMCs from HIV-1-infected donors on long-term ART (51). Thus, both TLR7 and TLR9 agonists have demonstrated NK cell-activating properties *ex vivo* which have subsequently been confirmed *in vivo* (69, 70, 73–75, 99). Broad activation of antiviral immunity evidenced by enhanced transcription of interferon-stimulated genes (ISGs) have been observed with TLR3, TLR7, and TLR9 agonists *in vivo* (69–71, 74).

Thus, both TLR7 and TLR9 agonists are very potent enhancers of innate immune effector functions and broad stimulators of adaptive immunity. The activation of antigen presenting immune cells in the presence of relevant antigens helps focusing the adaptive immune response to effectively target HIV-1. The parallel activation of immune effector cells could boost the clearance of infected cells and hence the ability to control the infection. These features of TLR agonists could be valuable assets in the development of a functional cure for HIV-1, based on the induction of immune control of the infection.

### Tissue Effects

As most TLRs are also expressed on epithelial cells throughout the body, TLR agonists may also affect resident cells in tissue (21). Our group investigated the effect of MGN1703 on gut mucosa epithelial cells, which express TLR9, using biopsies of the sigmoid colon from study participants of the short course MGN1703 treatment trial (74, 92). A global transcriptomic analysis showed that MGN1703 upregulated an ISG signature consistent with potent IFN-α induction. Furthermore, high levels of ISG proteins MX1 and ISG15 were detected by *in-situ* hybridization during MGN1703 stimulation in the epithelial cells. This induction was however not accompanied by excessive inflammation, evident by the lack of IFN-γ mRNA and absence of infiltrating neutrophils in the gut mucosa. These findings indicate that subcutaneously administered MGN1703 may have beneficial effects in the gut of infected individuals, but further investigations are needed to account for the exact mechanisms.

A sub-study to the 24-weeks MGN1703 treatment study (75) investigated the tissue-specific effects on lymph nodes (76). In LNMCs, augmented activation of pDCs, (CD86+ CD40+), NK cells (CD69+), and CD8+ effector memory T cells (HLA-DR+ CD38+) was detected and correlated with increased IFN levels and ISG15 expression. Interestingly, lymph node B cells displayed enhanced expression of activation-induced cytidine deaminase (AID) which is an essential enzyme in regulating B cell differentiation and somatic hypermutation. Consistently, a LNMC gene expression analysis showed markedly increased numbers of plasma cells post-MGN1703-treatment as well as increased levels of total IgG and subtypes IgG1, IgG2, and IgG3 antibodies. The induced antibodies showed specific neutralizing properties toward HIV-1 clade B and C, suggesting a possible autologous vaccinal effect of MGN1703. Finally, when assessing the architecture of the lymph nodes, it was demonstrated that the frequency of secondary follicles increased over the 24-weeks treatment period, suggestive of a restorative effect of the intervention.

In conclusion, these two studies from our group demonstrated broad immune enhancement and tissue restoration in the gut mucosa and lymph nodes following TLR9 agonist treatment. These results are encouraging for further clinical development as induction of potent immune responses in tissue compartments may be essential for the therapeutic success of HIV-1 cure strategies.

## TLR-Agonists in Combination With Other Therapeutics

### TLR Agonists as Vaccine Adjuvants

Developing a therapeutic HIV-1 vaccine has long been an approach to boost immune control of HIV-1. Moody et al. tested the adjuvant properties of three different TLR agonists, both alone and in combination, to HIV-1 envelope protein immunogens in rhesus macaques (100). They found that the combination of TLR9 agonist CpG ODN and TLR7/8 agonist R848 mixed with base adjuvant Span85-Tween 80-squalene (STS+oCpG+R848) elicited the most potent antibody response against HIV-1 envelope gp140 and V1V2-gp70 as measured by antibody titers. The elicited antibodies were superior in terms of neutralizing HIV-1 when comparing animals treated with STS+oCpG+R848 to those treated with STS alone. Additionally, STS+oCpG+R848-elicited antibodies had greater capacity for inducing ADCC against HIV-antigen-covered cells compared to antibodies from control animals. However, the elicited antibodies were short-lived with an estimated half-life of 8.5 weeks.

Kasturi et al. attempted to assess the issue of short-lived antibodies by altering the formulation of the adjuvant in a NHP preventive vaccine study (101). The adjuvants R848 and TLR4 agonist monophosphoryl lipid A were packed in nanoparticles (NP) and administered with soluble recombinant SIVmac239-derived envelope gp140 and Gag protein 55, together referred to as the protein-NP vaccine. This protein-NP vaccine elicited a durable antibody response present in both serum and mucosa of the rhesus macaques. Ten NHPs in each group received 1 of 4 combinations of immunogens and adjuvants (virus-like particles or soluble envelope combined with either gag + NP or alum). The vaccines were administered 4 times: at week 0, 8, 16, and 25. The protein-NP group had a significantly greater peak in antibody responses at week 27 with an envelope-specific IgG level of 680.77 μg/mL compared to 104.47, 78.71, and 19.81 μg/mL, in the three other groups. The protein-NP group also showed significantly higher levels of envelope-specific plasma cells in bone marrow and draining lymph nodes. In addition, the protein-NP group exhibited the most potent protection when all study animals were challenged 12 times intravaginally with low-dose SIV once weekly starting at week 41 after the first vaccination. Although the protection against infection was stronger in the protein-NP vaccination group, 5 of 10 NHPs still became infected after 10 challenges, stressing that although the NP administration improved the adjuvating effect of the TLR agonists, this was not sufficient to elicit complete protection.

Borducchi et al. conducted a study on SIV-infected Indian origin rhesus macaques, testing a novel therapeutic vaccine compound named Ad26/MVA. The vaccine comprised an adenovirus vector (Ad26) expressing SIV gag-pol-env, which was boosted by modified vaccinia Ankara (MVA), also expressing gag-pol-env and adjuvanted by TLR7 agonist GS-986 (93). Levels of IFN-γ were increased and CD4+ and CD8+ T cell activation was evident by increased CD69-expression in the vaccine group, indicating effective immune stimulation. Upon ART discontinuation, the 9 NHPs receiving the Ad26/MVA vaccine adjuvanted with GS-986 showed a 2.5-fold delay in time to viral rebound compared to sham controls. Additionally, three of the nine animals that initially appeared to have rebounded after ART cessation regained durable virologic control throughout 160 days. None of the three components administered alone were able to induce similar levels of control suggesting a synergistic effect of the TLR7 agonist and the Ad26/MVA vaccine.

In 2008, our group investigated the immunostimulatory effect of TLR9 agonist CpG 7909 as adjuvant to pneumococcal vaccines in a randomized double-blinded, placebo-controlled trial among 97 HIV-1 infected individuals (102). All study participants received a 7-valent pneumococcal conjugate vaccine (7vPnC) at 0 and 3 months and a 23 valent pneumococcal polysaccharide vaccine (PPV-23) at 9 months. The experimental group received 1 mg of CPG 7909 as adjuvant with each vaccine dose, while the control group received a placebo adjuvant. At 9 months, the proportion of high vaccine responders, defined as a 2-fold increase in IgG levels for at least 5 of 7 of the 7vPnC serotypes, was 48% in the experimental group (*n* = 48) compared to 25% in the control group (*n* = 49). CpG 7909 significantly enhanced the immunogenicity of the pneumococcal vaccine. Interestingly, 10 individuals in each study group were treatment-naïve. In the experimental group, the treatment-naïve individuals had a slight increase in plasma-HIV-1 RNA compared to the control treatment-naïve individuals which may reflect broad immune activation (102). A *post-hoc* analysis by Winckelmann et al. showed a significant reduction of 12.6% in the level of total HIV-1 DNA in PBMCs among individuals receiving CPG 7909 as adjuvant, compared to those receiving placebo (saline) (103). These findings triggered the further investigation into the TLR9 agonist MGN1703 as immunomodulator and latency reversing agent described in the sections above.

### TLR Agonists in Combination With bNAbs

Based on the immunological findings outlined above it has been proposed that administration of a TLR agonist in combination with bNAbs may enhance killing of infected cells by enhancing antibody-dependent effector mechanisms such as ADCC and ADCP (Figure 1). bNAbs function through three mechanisms: by direct viral neutralization, by opsonizing infected cells for immune mediated killing, and by activating the adaptive immune system via HIV-1-epitope-antibody-complexes (104–106).

Borducchi et al. investigated the effect of combining a bNAb (PGT121) with a TLR7 agonist (GS-9620) in a study with simian-human immunodeficiency virus (SHIV) infected rhesus macaques (99). The 44 NHPs were infected intrarectally with SHIV and ART was initiated at day 7 post-infection and continued for 96–104 weeks before bNAb and TLR7 agonist administration. ART was continued for another 16 weeks after the last administration of PGT121 before an ATI was initiated at week 130. By day 196 following ART cessation, only 6 of 11 NHPs in the PGT121+GS-9620 group had rebounded compared to 9 of 11 in the PGT121 group, 10 of 11 in the GS-9620 group, and 11 of 11 in the sham group. Of note, the viral reservoir measured as SHIV DNA in PBMCs was undetectable across all groups indicating that all animals hadvery small SHIV reservoirs, presumable due to the early initiation of ART. However, viral DNA was detectable in LNMCs and here, the PGT121+GS-9620 group had lower levels compared to the other groups. Subsequent anti-CD8α mediated CD8+ T-cell depletion in the non-rebounding NHPs failed to induce plasma viremia. Finally, adoptive transfer experiments where performed. PBMCs and LNMCs were first collected from the 2 PGT121+GS-9620 treated NHPs, who showed transcient rebound followed by durable virologic control. Cells were collected during the control phase of the ATI, but when transferred to SHIV-naïve NHPs, the cells induced infection. In contrast, adoptive transfer of cells from the 5 PGT121+GS-9620 treated NHPs who did not rebound upon ATI, did not cause infection in SHIV-naïve NHPs. These findings indicated that the viral SHIV reservoir was significantly reduced, maybe even completely eradicated in some animals by the combination of PGT121 and GS-9620 and have engouraged further investigations of this combination.

Along these lines, our group is performing an ongoing double-blinded randomized placebo controlled phase IIa clinical trial testing the TLR9 agonist MGN1703 and a combination of two bNAbs (3BNC117 and 101074) in HIV-1-infected donors on long-term ART, aiming to reduce the viral reservoir and induce immunological HIV-1 control (NCT03837756).

### TLR Agonists and Programmed Death-1 (PD-1) Inhibition

The PD-1 receptor is a marker of T cell exhaustion, which is upregulated following prolonged antigen-stimulation during infection (107) and in cancer (108). By alleviating this T cell exhaustion, immunotherapy with antibodies blocking the PD-1 receptor has dramatically improved the prognosis of many different types of cancers such as malignant melanoma and renal cell carcinoma (109–111). In HIV-1 cure research the hope is that treatment with anti-PD-1 antibodies might lead to a more efficient targeting of the latently infected cells by HIV-specific T cells.

Bekerman et al. tested the immunomodulatory effect of a chimeric anti-PD-1 antibody and the TLR7 agonist GS-9620 in chronically SIV infected rhesus macaques (94). In a four-arm controlled design, NHPs received 0.15 mg/kg GS-9620 by oral gavage every other week for a total of 10 administrations, alone or in combination with 10 mg/kg anti-PD-1 antibody. Upon ART cessation all of the 20 NHPs rebounded within 14 days with no delay compared to the placebo group. Assessment of the viral reservoir measured by total SIV DNA levels in PBMCs similarly showed no reduction. Proportions of IFN-γ- and IL-2-producing SIV-specific T cells also did not differ between the groups. Previous studies have found beneficial effects of PD-1 receptor inhibition during SIV infection in rhesus macaques, when intervening in viremic animals or shortly after ART initiation (112, 113). Importantly, Bekerman et al. administered the PD-1 receptor antibody and TLR7 agonist after 2 years of suppressive ART. This suggests that the possible therapeutic benefits of PD-1 receptor blockade may depend on timing of administration in relation to ART initiation.

## Concluding Remarks

Emerging preclinical and clinical studies strongly indicate that TLR agonists have great potential as immune boosting and priming agents in HIV-1 cure research. However, not all TLR agonist are created equally and so far, the most promising results have been observed following TLR7 and TLR9 administration. Expression patterns of TLRs in humans and the diverse response of the TLR subtypes are important factors to consider when assessing the potential clinical effect of TLR agonist treatment in HIV-1. Further investigation is needed into the physiological function of TLRs on immune cell subsets, differences in TLR expression in blood and tissue, as well as gender determined differences. TLR agonists should probably only be considered as moderately potent LRAs but while much interest in TLR agonists have evolved around their potential use as LRAs, we believe that pre-clinical and clinical findings demonstrate that the most important aspect of TLR agonists is their ability to enhance innate and adaptive immunity. This is underscored by the durable virologic control in the absence of ART achieved in NHPs by combining TLR agonists with AD26/MVA-vector SIV vaccines or bNAbs. Cross-priming of the CD8+ T cell response could be an important element of the antigen-dependent mechanisms seen with TLR agonist. Novel clinical trials testing these very concepts are now underway and their highly anticipated results will further inform the research field of the potential of TLR agonists as components in HIV-1 cure strategies.

## Author Contributions

JM, JG, and OS conceived and wrote the manuscript. All authors (+ JH and MT) contributed to manuscript revision and approved the submitted version.

## Conflict of Interest

The authors declare that the research was conducted in the absence of any commercial or financial relationships that could be construed as a potential conflict of interest.

## References

[1] Richman DD Margolis DM Delaney M Greene WC Hazuda D Pomerantz RJ. The challenge of finding a cure for HIV infection. Science. (2009) 323:1304–7. 10.1126/science.1165706 19265012

[2] Eisele E Siliciano RF. Redefining the viral reservoirs that prevent HIV-1 eradication. Immunity. (2012) 37:377–88. 10.1016/j.immuni.2012.08.010 22999944 PMC3963158

[3] Chun TW Carruth L Finzi D Shen X DiGiuseppe JA Taylor H. Quantification of latent tissue reservoirs and total body viral load in HIV-1 infection. Nature. (1997) 387:183–8. 10.1038/387183a0 9144289

[4] Finzi D Hermankova M Pierson T Carruth LM Buck C Chaisson RE. Identification of a reservoir for HIV-1 in patients on highly active antiretroviral therapy. Science. (1997) 278:1295–300. 10.1126/science.278.5341.1295 9360927

[5] Pierson T McArthur J Siliciano RF. Reservoirs for HIV-1: mechanisms for viral persistence in the presence of antiviral immune responses and antiretroviral therapy. Annu Rev Immunol. (2000) 18:665–708. 10.1146/annurev.immunol.18.1.665 10837072

[6] Chomont N El-Far M Ancuta P Trautmann L Procopio FA Yassine-Diab B. HIV reservoir size and persistence are driven by T cell survival and homeostatic proliferation. Nat Med. (2009) 15:893–900. 10.1038/nm.1972 19543283 PMC2859814

[7] Medzhitov R. Recognition of microorganisms and activation of the immune response. Nature. (2007) 449:819–26. 10.1038/nature06246 17943118

[8] Kumar H Kawai T Akira S. Pathogen recognition by the innate immune system. Int Rev Immunol. (2011) 30:16–34. 10.3109/08830185.2010.529976 21235323

[9] Zicari S Sessa L Cotugno N Ruggiero A Morrocchi E Concato C. Immune activation, inflammation, and Non-AIDS co-morbidities in HIV-infected patients under long-term ART. Viruses. (2019) 11:200. 10.3390/v11030200 30818749 PMC6466530

[10] Hemmi H Akira S. TLR signalling and the function of dendritic cells. Chem Immunol Allergy. (2005) 86:120–35. 10.1159/000086657 15976491

[11] Hornung V Rothenfusser S Britsch S Krug A Jahrsdorfer B Giese T. Quantitative expression of toll-like receptor 1-10 mRNA in cellular subsets of human peripheral blood mononuclear cells and sensitivity to CpG oligodeoxynucleotides. J Immunol. (2002) 168:4531–7. 10.4049/jimmunol.168.9.4531 11970999

[12] Kapsenberg ML. Dendritic-cell control of pathogen-driven T-cell polarization. Nat Rev Immunol. (2003) 3:984–93. 10.1038/nri1246 14647480

[13] Ochoa MT Legaspi AJ Hatziris Z Godowski PJ Modlin RL Sieling PA. Distribution of toll-like receptor 1 and toll-like receptor 2 in human lymphoid tissue. Immunology. (2003) 108:10–5. 10.1046/j.1365-2567.2003.01563.x 12519297 PMC1782859

[14] Akira S Uematsu S Takeuchi O. Pathogen recognition and innate immunity. Cell. (2006) 124:783–801. 10.1016/j.cell.2006.02.015 16497588

[15] Kanzler H Barrat FJ Hessel EM Coffman RL. Therapeutic targeting of innate immunity with Toll-like receptor agonists and antagonists. Nat Med. (2007) 13:552–9. 10.1038/nm1589 17479101

[16] Matsumoto M Seya T. TLR3: interferon induction by double-stranded RNA including poly(I:C). Adv Drug Deliv Rev. (2008) 60:805–12. 10.1016/j.addr.2007.11.005 18262679

[17] Akira SB Hartmann G Toll Like Receptors (TLRs) and Innate Immunity. Berlin; Heidelberg: Springer-Verlag (2008).

[18] Kawai T Akira S. The role of pattern-recognition receptors in innate immunity: update on Toll-like receptors. Nat Immunol. (2010) 11:373–84. 10.1038/ni.1863 20404851

[19] Petes C Odoardi N Gee K. The toll for trafficking: toll-like receptor 7 delivery to the endosome. Front Immunol. (2017) 8:1075. 10.3389/fimmu.2017.01075 28928743 PMC5591332

[20] Tartey S Takeuchi O. Pathogen recognition and Toll-like receptor targeted therapeutics in innate immune cells. Int Rev Immunol. (2017) 36:57–73. 10.1080/08830185.2016.1261318 28060562

[21] McClure R Massari P. TLR-dependent human mucosal epithelial cell responses to microbial pathogens. Front Immunol. (2014) 5:386. 10.3389/fimmu.2014.00386 25161655 PMC4129373

[22] Iwasaki A Medzhitov R. Toll-like receptor control of the adaptive immune responses. Nat Immunol. (2004) 5:987–95. 10.1038/ni1112 15454922

[23] Kawasaki T Kawai T. Toll-like receptor signaling pathways. Front Immunol. (2014) 5:461. 10.3389/fimmu.2014.00461 25309543 PMC4174766

[24] Verstak B Nagpal K Bottomley SP Golenbock DT Hertzog PJ Mansell A. MyD88 adapter-like (Mal)/TIRAP interaction with TRAF6 is critical for TLR2- and TLR4-mediated NF-kappaB proinflammatory responses. J Biol Chem. (2009) 284:24192–203. 10.1074/jbc.M109.023044 19592497 PMC2782013

[25] Macedo AB Novis CL Bosque A. Targeting cellular and tissue HIV reservoirs with toll-like receptor agonists. Front Immunol. (2019) 10:2450. 10.3389/fimmu.2019.02450 31681325 PMC6804373

[26] Altfeld M Gale M Jr Innate immunity against HIV-1 infection. Nat Immunol. (2015) 16:554–62. 10.1038/ni.3157 25988887

[27] Deeks SG Lewin SR Ross AL Ananworanich J Benkirane M Cannon P. International AIDS Society global scientific strategy: towards an HIV cure 2016. Nat Med. (2016) 22:839–50. 10.1038/nm.4108 27400264 PMC5322797

[28] Sadowski I Hashemi FB. Strategies to eradicate HIV from infected patients: elimination of latent provirus reservoirs. Cell Mol Life Sci. (2019) 76:3583–600. 10.1007/s00018-019-03156-8 31129856 PMC6697715

[29] Deeks SG. HIV: Shock and kill. Nature. (2012) 487:439–40. 10.1038/487439a 22836995

[30] Sengupta S Siliciano RF. Targeting the latent reservoir for HIV-1. Immunity. (2018) 48:872–95. 10.1016/j.immuni.2018.04.030 29768175 PMC6196732

[31] Elliott JH Wightman F Solomon A Ghneim K Ahlers J Cameron MJ. Activation of HIV transcription with short-course vorinostat in HIV-infected patients on suppressive antiretroviral therapy. PLoS Pathog. (2014) 10:e1004473. 10.1371/journal.ppat.1004473 25393648 PMC4231123

[32] Rasmussen TA Tolstrup M Brinkmann CR Olesen R Erikstrup C Solomon A. Panobinostat, a histone deacetylase inhibitor, for latent-virus reactivation in HIV-infected patients on suppressive antiretroviral therapy: a phase 1/2, single group, clinical trial. Lancet HIV. (2014) 1:e13–21. 10.1016/S2352-3018(14)70014-1 26423811

[33] Sogaard OS Graversen ME Leth S Olesen R Brinkmann CR Nissen SK. The depsipeptide romidepsin reverses HIV-1 latency *in vivo*. PLoS Pathog. (2015) 11:e1005142. 10.1371/journal.ppat.1005142 26379282 PMC4575032

[34] Karn J Stoltzfus CM. Transcriptional and posttranscriptional regulation of HIV-1 gene expression. Cold Spring Harb Perspect Med. (2012) 2:a006916. 10.1101/cshperspect.a006916 22355797 PMC3281586

[35] Eckschlager T Plch J Stiborova M Hrabeta J. Histone deacetylase inhibitors as anticancer drugs. Int J Mol Sci. (2017) 18:1414. 10.3390/ijms18071414 28671573 PMC5535906

[36] Leth S Schleimann MH Nissen SK Hojen JF Olesen R Graversen ME. Combined effect of Vacc-4x, recombinant human granulocyte macrophage colony-stimulating factor vaccination, and romidepsin on the HIV-1 reservoir (REDUC): a single-arm, phase 1B/2A trial. Lancet HIV. (2016) 3:e463–472. 10.1016/S2352-3018(16)30055-8 27658863

[37] Bashiri K Rezaei N Nasi M Cossarizza A. The role of latency reversal agents in the cure of HIV: a review of current data. Immunol Lett. (2018) 196:135–9. 10.1016/j.imlet.2018.02.004 29427743

[38] Kulkosky J Sullivan J Xu Y Souder E Hamer DH Pomerantz RJ. Expression of latent HAART-persistent HIV type 1 induced by novel cellular activating agents. AIDS Res Hum Retroviruses. (2004) 20:497–505. 10.1089/088922204323087741 15186524

[39] Gutierrez C Serrano-Villar S Madrid-Elena N Perez-Elias MJ Martin ME Barbas C. Bryostatin-1 for latent virus reactivation in HIV-infected patients on antiretroviral therapy. AIDS. (2016) 30:1385–92. 10.1097/QAD.0000000000001064 26891037

[40] Elliott JH McMahon JH Chang CC Lee SA Hartogensis W Bumpus N. Short-term administration of disulfiram for reversal of latent HIV infection: a phase 2 dose-escalation study. Lancet HIV. (2015) 2:e520–9. 10.1016/S2352-3018(15)00226-X 26614966 PMC5108570

[41] Saleh S Wightman F Ramanayake S Alexander M Kumar N Khoury G. Expression and reactivation of HIV in a chemokine induced model of HIV latency in primary resting CD4+ T cells. Retrovirology. (2011) 8:80. 10.1186/1742-4690-8-80 21992606 PMC3215964

[42] Jiang G Nguyen D Archin NM Yukl SA Mendez-Lagares G Tang Y. HIV latency is reversed by ACSS2-driven histone crotonylation. J Clin Invest. (2018) 128:1190–8. 10.1172/JCI98071 29457784 PMC5824862

[43] Lisziewicz J Sun D Weichold FF Thierry AR Lusso P Tang J. Antisense oligodeoxynucleotide phosphorothioate complementary to Gag mRNA blocks replication of human immunodeficiency virus type 1 in human peripheral blood cells. Proc Natl Acad Sci USA. (1994) 91:7942–6. 10.1073/pnas.91.17.7942 8058738 PMC44520

[44] Sereni D Tubiana R Lascoux C Katlama C Taulera O Bourque A. Pharmacokinetics and tolerability of intravenous trecovirsen (GEM 91), an antisense phosphorothioate oligonucleotide, in HIV-positive subjects. J Clin Pharmacol. (1999) 39:47–54. 10.1177/00912709922007552 9987700

[45] Krieg AM. CpG motifs in bacterial DNA and their immune effects. Annu Rev Immunol. (2002) 20:709–60. 10.1146/annurev.immunol.20.100301.064842 11861616

[46] Agrawal S Martin RR. Was induction of HIV-1 through TLR9? J Immunol. (2003) 171:1621; author reply 1621-1622. 10.4049/jimmunol.171.4.1621 12902456

[47] Equils O Schito ML Karahashi H Madak Z Yarali A Michelsen KS. Toll-like receptor 2 (TLR2) and TLR9 signaling results in HIV-long terminal repeat trans-activation and HIV replication in HIV-1 transgenic mouse spleen cells: implications of simultaneous activation of TLRs on HIV replication. J Immunol. (2003) 170:5159–64. 10.4049/jimmunol.170.10.5159 12734363

[48] Novis CL Archin NM Buzon MJ Verdin E Round JL Lichterfeld M. Reactivation of latent HIV-1 in central memory CD4(+) T cells through TLR-1/2 stimulation. Retrovirology. (2013) 10:119. 10.1186/1742-4690-10-119 24156240 PMC3826617

[49] Kaur J Tsai A Kukolj G Cihlar T Murry J Pattern recognition receptor agonists induce HIV in ART suppressed HIV+ donor cells. CROI 2018 abstract #321. Boston, MA (2018).

[50] Thibault S Imbeault M Tardif MR Tremblay MJ. TLR5 stimulation is sufficient to trigger reactivation of latent HIV-1 provirus in T lymphoid cells and activate virus gene expression in central memory CD4+ T cells. Virology. (2009) 389:20–5. 10.1016/j.virol.2009.04.019 19447460

[51] Tsai A Irrinki A Kaur J Cihlar T Kukolj G Sloan DD. Toll-Like Receptor 7 Agonist GS-9620 Induces HIV Expression and HIV-Specific Immunity in Cells from HIV-Infected Individuals on Suppressive Antiretroviral Therapy. J Virol. (2017) 91. 10.1128/JVI.02166-16 28179531 PMC5375698

[52] Bam RA Hansen D Irrinki A Mulato A Jones GS Hesselgesser J. TLR7 Agonist GS-9620 Is a Potent Inhibitor of Acute HIV-1 Infection in Human Peripheral Blood Mononuclear Cells. Antimicrob Agents Chemother. (2016) 61:e01369–16. 10.1128/AAC.01369-16 27799218 PMC5192112

[53] Offersen R Nissen SK Rasmussen TA Ostergaard L Denton PW Sogaard OS. A novel toll-like receptor 9 agonist, MGN1703, enhances HIV-1 transcription and NK cell-mediated inhibition of HIV-1-infected autologous CD4+ T cells. J Virol. (2016) 90:4441–53. 10.1128/JVI.00222-16 26889036 PMC4836316

[54] Chaturvedi A Pierce SK. How location governs toll-like receptor signaling. Traffic. (2009) 10:621–8. 10.1111/j.1600-0854.2009.00899.x 19302269 PMC2741634

[55] Caron G Duluc D Fremaux I Jeannin P David C Gascan H. Direct stimulation of human T cells via TLR5 and TLR7/8: flagellin and R-848 up-regulate proliferation and IFN-gamma production by memory CD4+ T cells. J Immunol. (2005) 175:1551–7. 10.4049/jimmunol.175.3.1551 16034093

[56] Krieg AM. Development of TLR9 agonists for cancer therapy. J Clin Invest. (2007) 117:1184–94. 10.1172/JCI31414 17476348 PMC1857270

[57] Schmidt M Hagner N Marco A Konig-Merediz SA Schroff M Wittig B. Design and structural requirements of the potent and safe TLR-9 agonistic immunomodulator MGN1703. Nucleic Acid Ther. (2015) 25:130–40. 10.1089/nat.2015.0533 25826686 PMC4440985

[58] Schlaepfer E Speck RF. TLR8 activates HIV from latently infected cells of myeloid-monocytic origin directly via the MAPK pathway and from latently infected CD4+ T cells indirectly via TNF-alpha. J Immunol. (2011) 186:4314–24. 10.4049/jimmunol.1003174 21357269

[59] Macedo AB Novis CL De Assis CM Sorensen ES Moszczynski P Huang SH. Dual TLR2 and TLR7 agonists as HIV latency-reversing agents. JCI Insight. (2018) 3:e122673. 10.1172/jci.insight.122673 30282829 PMC6237480

[60] Stoszko M Ne E Abner E Mahmoudi T. A broad drug arsenal to attack a strenuous latent HIV reservoir. Curr Opin Virol. (2019) 38:37–53. 10.1016/j.coviro.2019.06.001 31323521

[61] Telwatte S Moron-Lopez S Aran D Kim P Hsieh C Joshi S. Heterogeneity in HIV and cellular transcription profiles in cell line models of latent and productive infection: implications for HIV latency. Retrovirology. (2019) 16:32. 10.1186/s12977-019-0494-x 31711503 PMC6849327

[62] Ketloy C Engering A Srichairatanakul U Limsalakpetch A Yongvanitchit K Pichyangkul S. Expression and function of Toll-like receptors on dendritic cells and other antigen presenting cells from non-human primates. Vet Immunol Immunopathol. (2008) 125:18–30. 10.1016/j.vetimm.2008.05.001 18571243

[63] Gujer C Sundling C Seder RA Karlsson Hedestam GB Lore K. Human and rhesus plasmacytoid dendritic cell and B-cell responses to Toll-like receptor stimulation. Immunology. (2011) 134:257–69. 10.1111/j.1365-2567.2011.03484.x 21977996 PMC3209566

[64] Caskey M Lefebvre F Filali-Mouhim A Cameron MJ Goulet JP Haddad EK. Synthetic double-stranded RNA induces innate immune responses similar to a live viral vaccine in humans. J Exp Med. (2011) 208:2357–66. 10.1084/jem.20111171 22065672 PMC3256967

[65] Cheng L Zhang Z Li G Li F Wang L Zhang L. Human innate responses and adjuvant activity of TLR ligands *in vivo* in mice reconstituted with a human immune system. Vaccine. (2017) 35:6143–53. 10.1016/j.vaccine.2017.09.052 28958808 PMC5641266

[66] Cheng L Wang Q Li G Banga R Ma J Yu H. TLR3 agonist and CD40-targeting vaccination induces immune responses and reduces HIV-1 reservoirs. J Clin Invest. (2018) 128:4387–96. 10.1172/JCI99005 30148455 PMC6159955

[67] Kyi C Roudko V Sabado R Saenger Y Loging W Mandeli J. Therapeutic Immune Modulation against Solid Cancers with Intratumoral Poly-ICLC: A Pilot Trial. Clin Cancer Res. (2018) 24:4937–48. 10.1158/1078-0432.CCR-17-1866 29950349 PMC6191332

[68] Wittig B Schmidt M Scheithauer W Schmoll HJ. MGN1703, an immunomodulator and toll-like receptor 9 (TLR-9) agonist: from bench to bedside. Crit Rev Oncol Hematol. (2015) 94:31–44. 10.1016/j.critrevonc.2014.12.002 25577571

[69] Lim SY Osuna CE Hraber PT Hesselgesser J Gerold JM Barnes TL. TLR7 agonists induce transient viremia and reduce the viral reservoir in SIV-infected rhesus macaques on antiretroviral therapy. Sci Transl Med. (2018) 10:eaao4521. 10.1126/scitranslmed.aao4521 29720451 PMC5973480

[70] Del Prete GQ Alvord WG Li Y Deleage C Nag M Oswald K. TLR7 agonist administration to SIV-infected macaques receiving early initiated cART does not induce plasma viremia. JCI Insight. (2019) 4:e127717. 10.1172/jci.insight.127717 31167974 PMC6629134

[71] Saxena M Sabado RL La Mar M Mohri H Salazar AM Dong H. Poly-ICLC, a TLR3 agonist, induces transient innate immune responses in patients with treated hiv-infection: a randomized double-blinded placebo controlled trial. Front Immunol. (2019) 10:725. 10.3389/fimmu.2019.00725 31024557 PMC6467168

[72] Salazar AM Erlich RB Mark A Bhardwaj N Herberman RB. Therapeutic in situ autovaccination against solid cancers with intratumoral poly-ICLC: case report, hypothesis, and clinical trial. Cancer Immunol Res. (2014) 2:720–4. 10.1158/2326-6066.CIR-14-0024 24801836

[73] Riddler S Vesatolimod (GS-9620) is Safe and Pharmacodynamically Active in HIV-Infected Individuals. Mexico City: IAS 2019 oral #WEAA0304 (2019).

[74] Vibholm L Schleimann MH Hojen JF Benfield T Offersen R Rasmussen K. Short-course toll-like receptor 9 agonist treatment impacts innate immunity and plasma viremia in individuals with human immunodeficiency virus infection. Clin Infect Dis. (2017) 64:1686–95. 10.1093/cid/cix201 28329286 PMC5849129

[75] Vibholm LK Konrad CV Schleimann MH Frattari G Winckelmann A Klastrup V. Effects of 24-week toll-like receptor 9 agonist treatment in HIV type 1+ individuals. AIDS. (2019) 33:1315–25. 10.1097/QAD.0000000000002213 30932955

[76] Schleimann MH Kobbero ML Vibholm LK Kjaer K Giron LB Busman-Sahay K. TLR9 agonist MGN1703 enhances B cell differentiation and function in lymph nodes. EBioMedicine. (2019) 45:328–40. 10.1016/j.ebiom.2019.07.005 31300344 PMC6642412

[77] Kumar A Abbas W Herbein G. HIV-1 latency in monocytes/macrophages. Viruses. (2014) 6:1837–60. 10.3390/v6041837 24759213 PMC4014723

[78] Kandathil AJ Sugawara S Balagopal A Are T cells the only HIV-1 reservoir? Retrovirology. (2016) 13:86 10.1186/s12977-016-0323-4 27998285 PMC5175311

[79] Sheth PM Kovacs C Kemal KS Jones RB Raboud JM Pilon R. Persistent HIV RNA shedding in semen despite effective antiretroviral therapy. AIDS. (2009) 23:2050–4. 10.1097/QAD.0b013e3283303e04 19710596

[80] Yukl SA Shergill AK Ho T Killian M Girling V Epling L. The distribution of HIV DNA and RNA in cell subsets differs in gut and blood of HIV-positive patients on ART: implications for viral persistence. J Infect Dis. (2013) 208:1212–20. 10.1093/infdis/jit308 23852128 PMC3778964

[81] Costiniuk CT Jenabian MA. The lungs as anatomical reservoirs of HIV infection. Rev Med Virol. (2014) 24:35–54. 10.1002/rmv.1772 24151040

[82] Sewald X Ladinsky MS Uchil PD Beloor J Pi R Herrmann C. Retroviruses use CD169-mediated trans-infection of permissive lymphocytes to establish infection. Science. (2015) 350:563–7. 10.1126/science.aab2749 26429886 PMC4651917

[83] Ganor Y Real F Sennepin A Dutertre CA Prevedel L Xu L. HIV-1 reservoirs in urethral macrophages of patients under suppressive antiretroviral therapy. Nat Microbiol. (2019) 4:633–44. 10.1038/s41564-018-0335-z 30718846

[84] Wallet C De Rovere M Van Assche J Daouad F De Wit S Gautier V. Microglial cells: the main HIV-1 reservoir in the brain. Front Cell Infect Microbiol. (2019) 9:362. 10.3389/fcimb.2019.00362 31709195 PMC6821723

[85] Rajasekaran N Chester C Yonezawa A Zhao X Kohrt HE. Enhancement of antibody-dependent cell mediated cytotoxicity: a new era in cancer treatment. Immunotargets Ther. (2015) 4:91–100. 10.2147/ITT.S61292 27471715 PMC4918249

[86] Satoh T Akira S Toll-Like Receptor Signaling and Its Inducible Proteins. Microbiol Spectr. (2016) 4 10.1128/microbiolspec.MCHD-0040-2016 28084212

[87] Longhi MP Trumpfheller C Idoyaga J Caskey M Matos I Kluger C. Dendritic cells require a systemic type I interferon response to mature and induce CD4+ Th1 immunity with poly IC as adjuvant. J Exp Med. (2009) 206:1589–602. 10.1084/jem.20090247 19564349 PMC2715098

[88] Tai Y Wang Q Korner H Zhang L Wei W. Molecular mechanisms of t cells activation by dendritic cells in autoimmune diseases. Front Pharmacol. (2018) 9:642. 10.3389/fphar.2018.00642 29997500 PMC6028573

[89] Hervas-Stubbs S Perez-Gracia JL Rouzaut A Sanmamed MF Le Bon A Melero I. Direct effects of type I interferons on cells of the immune system. Clin Cancer Res. (2011) 17:2619–27. 10.1158/1078-0432.CCR-10-1114 21372217

[90] Tolstrup M. TLR Agonists and Latency Reversal: Can They Both Shock and Kill? Amsterdam: AIDS 2018 oral #WESY0905. (2018).

[91] Speiser DE Lienard D Rufer N Rubio-Godoy V Rimoldi D Lejeune F. Rapid and strong human CD8+ T cell responses to vaccination with peptide, IFA, and CpG oligodeoxynucleotide 7909. J Clin Invest. (2005) 115:739–46. 10.1172/JCI23373 15696196 PMC546459

[92] Krarup AR Abdel-Mohsen M Schleimann MH Vibholm L Engen PA Dige A. The TLR9 agonist MGN1703 triggers a potent type I interferon response in the sigmoid colon. Mucosal Immunol. (2018) 11:449–61. 10.1038/mi.2017.59 28766555 PMC5796873

[93] Borducchi EN Cabral C Stephenson KE Liu J Abbink P Ng'ang'a D. Ad26/MVA therapeutic vaccination with TLR7 stimulation in SIV-infected rhesus monkeys. Nature. (2016) 540:284–7. 10.1038/nature20583 27841870 PMC5145754

[94] Bekerman E Hesselgesser J Carr B Nagel M Hung M Wang A. PD-1 Blockade and TLR7 activation lack therapeutic benefit in chronic simian immunodeficiency virus-infected macaques on antiretroviral therapy. Antimicrob Agents Chemother. (2019) 63:e01163–19. 10.1128/AAC.01163-19 31501143 PMC6811450

[95] Cassatella MA Anegon I Cuturi MC Griskey P Trinchieri G Perussia B. Fc gamma R(CD16) interaction with ligand induces Ca2+ mobilization and phosphoinositide turnover in human natural killer cells. Role of Ca2+ in Fc gamma R(CD16)-induced transcription and expression of lymphokine genes. J Exp Med. (1989) 169:549–67. 10.1084/jem.169.2.549 2536067 PMC2189210

[96] Molgora M Supino D Mavilio D Santoni A Moretta L Mantovani A. The yin-yang of the interaction between myelomonocytic cells and NK cells. Scand J Immunol. (2018) 88:e12705. 10.1111/sji.12705 30048003 PMC6485394

[97] Mikulak J Oriolo F Zaghi E Di Vito C Mavilio D. Natural killer cells in HIV-1 infection and therapy. AIDS. (2017) 31:2317–30. 10.1097/QAD.0000000000001645 28926399 PMC5892189

[98] Tay MZ Wiehe K Pollara J. Antibody-dependent cellular phagocytosis in antiviral immune responses. Front Immunol. (2019) 10:332. 10.3389/fimmu.2019.00332 30873178 PMC6404786

[99] Borducchi EN Liu J Nkolola JP Cadena AM Yu WH Fischinger S. Antibody and TLR7 agonist delay viral rebound in SHIV-infected monkeys. Nature. (2018) 563:360–4. 10.1038/s41586-018-0600-6 30283138 PMC6237629

[100] Moody MA Santra S Vandergrift NA Sutherland LL Gurley TC Drinker MS. Toll-like receptor 7/8 (TLR7/8) and TLR9 agonists cooperate to enhance HIV-1 envelope antibody responses in rhesus macaques. J Virol. (2014) 88:3329–39. 10.1128/JVI.03309-13 24390332 PMC3957956

[101] Kasturi SP Kozlowski PA Nakaya HI Burger MC Russo P Pham M. Adjuvanting a Simian Immunodeficiency Virus Vaccine with Toll-Like Receptor Ligands Encapsulated in Nanoparticles Induces Persistent Antibody Responses and Enhanced Protection in TRIM5α Restrictive Macaques. J Virol. (2017) 91:e01844–16. 10.1128/JVI.01844-16 27928002 PMC5286877

[102] Sogaard OS Lohse N Harboe ZB Offersen R Bukh AR Davis HL. Improving the immunogenicity of pneumococcal conjugate vaccine in HIV-infected adults with a toll-like receptor 9 agonist adjuvant: a randomized, controlled trial. Clinical Infectious Diseases. (2010) 51:42–50. 10.1086/653112 20504165

[103] Winckelmann AA Munk-Petersen LV Rasmussen TA Melchjorsen J Hjelholt TJ Montefiori D. Administration of a toll-like receptor 9 agonist decreases the proviral reservoir in virologically suppressed HIV-infected patients. PLoS ONE. (2013) 8:e62074. 10.1371/journal.pone.0062074 23637967 PMC3637371

[104] Bournazos S Klein F Pietzsch J Seaman MS Nussenzweig MC Ravetch JV. Broadly neutralizing anti-HIV-1 antibodies require Fc effector functions for *in vivo* activity. Cell. (2014) 158:1243–53. 10.1016/j.cell.2014.08.023 25215485 PMC4167398

[105] Schoofs T Klein F Braunschweig M Kreider EF Feldmann A Nogueira L. HIV-1 therapy with monoclonal antibody 3BNC117 elicits host immune responses against HIV-1. Science. (2016) 352:997–1001. 10.1126/science.aaf0972 27199429 PMC5151174

[106] Caskey M Klein F Nussenzweig MC. Broadly neutralizing anti-HIV-1 monoclonal antibodies in the clinic. Nat Med. (2019) 25:547–53. 10.1038/s41591-019-0412-8 30936546 PMC7322694

[107] Barber DL Wherry EJ Masopust D Zhu B Allison JP Sharpe AH. Restoring function in exhausted CD8 T cells during chronic viral infection. Nature. (2006) 439:682–7. 10.1038/nature04444 16382236

[108] Ahmadzadeh M Johnson LA Heemskerk B Wunderlich JR Dudley ME White DE. Tumor antigen-specific CD8 T cells infiltrating the tumor express high levels of PD-1 and are functionally impaired. Blood. (2009) 114:1537–44. 10.1182/blood-2008-12-195792 19423728 PMC2927090

[109] Weinstock M McDermott D. Targeting PD-1/PD-L1 in the treatment of metastatic renal cell carcinoma. Ther Adv Urol. (2015) 7:365–77. 10.1177/1756287215597647 26622321 PMC4647139

[110] Munhoz RR Postow MA. Clinical Development of PD-1 in Advanced Melanoma. Cancer J. (2018) 24:7–14. 10.1097/PPO.0000000000000299 29360722 PMC5819364

[111] Wu X Gu Z Chen Y Chen B Chen W Weng L. Application of PD-1 Blockade in Cancer Immunotherapy. Comput Struct Biotechnol J. (2019) 17:661–74. 10.1016/j.csbj.2019.03.006 31205619 PMC6558092

[112] Velu V Titanji K Zhu B Husain S Pladevega A Lai L. Enhancing SIV-specific immunity *in vivo* by PD-1 blockade. Nature. (2009) 458:206–10. 10.1038/nature07662 19078956 PMC2753387

[113] Mylvaganam GH Chea LS Tharp GK Hicks S Velu V Iyer SS. Combination anti-PD-1 antiretroviral therapy provides therapeutic benefit against SIV. JCI Insight. (2018) 3:e122940. 10.1172/jci.insight.122940 30232277 PMC6237231

